# Parallelistic Convolution Neural Network Approach for Brain Tumor Diagnosis

**DOI:** 10.3390/diagnostics12102484

**Published:** 2022-10-13

**Authors:** Goodness Temofe Mgbejime, Md Altab Hossin, Grace Ugochi Nneji, Happy Nkanta Monday, Favour Ekong

**Affiliations:** 1 School of Computer Science and Engineering, University of Electronic Science and Technology of China, Chengdu 611731, China; 2 School of Innovation and Entrepreneurship, Chengdu University, Chengdu 610106, China; 3 Department of Computing, Oxford Brookes College of Chengdu University of Technology, Chengdu 610059, China; 4 Deep Learning and Intelligent Computing Lab, HACE SOFTTECH, Lagos 102241, Nigeria; 5 School of Information and Software Engineering, University of Electronic Science and Technology of China, Chengdu 611731, China

**Keywords:** brain tumor, disease diagnosis, deep learning, MRI, medical imaging

## Abstract

Today, Magnetic Resonance Imaging (MRI) is a prominent technique used in medicine, produces a significant and varied range of tissue contrasts in each imaging modalities, and is frequently employed by medical professionals to identify brain malignancies. With brain tumor being a very deadly disease, early detection will help increase the likelihood that the patient will receive the appropriate medical care leading to either a full elimination of the tumor or the prolongation of the patient’s life. However, manually examining the enormous volume of magnetic resonance imaging (MRI) images and identifying a brain tumor or cancer is extremely time-consuming and requires the expertise of a trained medical expert or brain doctor to manually detect and diagnose brain cancer using multiple Magnetic Resonance images (MRI) with various modalities. Due to this underlying issue, there is a growing need for increased efforts to automate the detection and diagnosis process of brain tumor without human intervention. Another major concern most research articles do not consider is the low quality nature of MRI images which can be attributed to noise and artifacts. This article presents a Contrast Limited Adaptive Histogram Equalization (CLAHE) algorithm to precisely handle the problem of low quality MRI images by eliminating noisy elements and enhancing the visible trainable features of the image. The enhanced image is then fed to the proposed PCNN to learn the features and classify the tumor using sigmoid classifier. To properly train the model, a publicly available dataset is collected and utilized for this research. Additionally, different optimizers and different values of dropout and learning rates are used in the course of this study. The proposed PCNN with Contrast Limited Adaptive Histogram Equalization (CLAHE) algorithm achieved an accuracy of 98.7%, sensitivity of 99.7%, and specificity of 97.4%. In comparison with other state-of-the-art brain tumor methods and pre-trained deep transfer learning models, the proposed PCNN model obtained satisfactory performance.

## 1. Introduction

The human brain has billions of neurons, the primary functions of which are organ control and information processing. The complexity of the human brain exceeds what is currently understood. The cerebrum, brain stem, and cerebellum are the three components that make up the human brain [1]. The skull acts as a safeguard surrounding these areas, preventing external threats from harming or damaging the brain. The skull however, is not very effective in safeguarding the brain from internal neurological causes. Tumors are one of the most harmful internal causes because they affect the brain’s cells at the cellular level and result to patient death [2]. According to research, early tumor detection and treatment have a significant positive impact on patients’ survival. As a result, brain imaging is crucial in the process of diagnosing various injuries and malignancies. There are several imaging technologies, computed tomography (CT), X-ray image and magnetic resonance imaging (MRI). The rapid development of digital technology and the improvement in image technology are directly related. The most cutting-edge imaging technique is Magnetic Resonance Imaging (MRI) and it is used to visualize and picture the internal parts of the body [3]. The MRI scanner may provide precise anatomical data on soft tissues in the various parts of the human body with the help of a strong magnet. MRI has already demonstrated efficacy in identifying cardiac problems and brain diseases. MRI technology creates images of the brain tissues using radio waves, magnetic fields, and other technologies. The factors of signal frequency and magnetic field intensity are used to produce 4 distinct types of modalities or images. These include longitudinal relation time- weighted (T1-weighted), T1-contrasted, transverse relaxation time- weighted (T2-weighted) and fluid-attenuated inversion recovery (Flair) [4]. Each of the 4 distinct modalities in these images may be identified by way of color, contrast, and several other elements. For instance, T1 represents the darker areas of the image, T2 the brighter areas, and Flair represents water and macromolecules. The MRI image may be utilized effectively to finely differentiate between these numerous states of the human body tissues due to the variations in the physical features of these states (such as bleeding, edema, inflammation, and tumors) [5]. Even though doctors and clinical technicians have vast training and knowledge to detect the presence or absence of a tumor in a MRI Image, a definitive diagnosis requires a great deal of effort and time.In addition to taking more time and effort, if the doctor needs to review a lot of MRI images to work on within a short period of time, there may be a higher chance of diagnosis error. Researchers have turned to computer-aided diagnostic systems based on image recognition and classification to overcome these issues. One such method is Deep Learning also known as DL, which allows images to be automatically identified with a high degree of accuracy [6]. Additionally, these computer-aided methods have the ability to extract features from these MRI images which are then used to divide MRI scans into benign and malignant groups without the need for human involvement. One of the most commonly used DL methods is convolutional neural networks (CNN), which has a wide range of applications in the field of medical diagnostics [7].

Several deep learning models are so complicated especially very deep networks which has necessitated the creation of convolution layers arranged in parallel with multiple filters stacked into different blocks. Since models are chosen based on the nature of the data and problem requirement, the proposed method combines multiple filters to address this problem. This technique is proposed to assist very deep convolution neural network models overcome their flaws of vanishing gradient problem and solidify their capability by aggregating the features extracted from different filters in a fused manner. This proposed algorithm reduces generalization error, vanishing gradient problem, and minimizes prediction variance. Thus, the goal of this study is to analyze the performance of deep convolution neural network with multiple connected filters for brain tumor classification.

The main contributions of this paper are as follows:

- We apply contrast limited adaptive histogram equalization algorithm for the elimination of noisy contents and enhancing the visual trainable features of these MRI brain images.
- We implemented a stacked connections of convolution layers with multiple filters in parallel to learn the enhanced MRI images and then classify the brain tumor using sigmoid classifier.

The proposed network can automatically classify benign and malignant brain MRI images. In this study, We evaluate the performance of the proposed model and other state-of-the-art methods.

The subsequent sections of this paper is organized in the following manner. Section 2 presents prior studies related to brain tumor diagnosis and motivation for the research. Section 3 presents the methodology adopted for this study. Experimental results are presented in Section 4 while Section 5 presents the discussion. Section 6 concludes this study and finally, Section 7 presents the limitation of this study with recommendation for future research directions.

## 2. Related Works

Brain tumor classification is an extremely challenging process that requires the correct expertise, training, aptitude, and analysis procedures to detect [8]. Positron Emission Tomography (PET), Computed Tomography (CT), and Magnetic Resonance Imaging are the three imaging modalities that physicians utilize to identify brain malignancies (MRI). Radio waves, strong magnets, and software for computation are all used in MRI to record the interior intricacies of the brain. Because of the characteristics of tissue relaxation (T1 and T2), MRI offers superior contrast, brightness, and image details when compared to the other techniques, which is why physicians recommend them as a diagnostic tool [9].

Numerous automated approaches have recently been suggested and created by researchers in order to categorize brain cancers using MRI data [10]. The authors in [11] used a pre-processing step of a proposed feature-based automated approach for classifying brain MRI images. Along with Random Forests (RFs), block-based feature extraction is suggested for the binary categorization of MRI images into Benign and malignant using the dataset from BraTS 2015. Specificity, sensitivity, Missed Alarm (MA), accuracy, and False Alarm are metrics used to verify the results (FA). The findings showed a 94% sensitivity and specificity, 95% accuracy with a 1% error rate, and a 3% spuriously tumorous classification rate. Another author employed in the segmentation and categorization of brain tumors with several classifiers found in WEKA. Thus, the findings revealed that Random Forest (RF) classifier performed better than all other classifiers for the chosen characteristics with an averaging 80.86% accuracy [12].

A CAD system including image segmentation, feature extraction, and multiclass classification of six kinds of brain tumors was created in 2013 by Sachdeva et al. [13]. Using artificial neural networks in three separate studies, the total classification accuracy was found to be 85%. Support vector machine (SVM), a machine learning approach was utilized to classify benign and malignant cancers achieving 91.5% accuracy, 90.8% sensitivity, and 94.7% specificity [14]. Prior to executing the classification operation, extracting the important features from the provided data is important. To address this, a hybrid feature extraction technique using a regularized extreme learning machine (RELM) was presented in [15]. Using fully connected neural networks and convolutional neural networks (CNN), Paul et al. [16] created a generalized technique for classifying brain tumors obtaining 91.43% accuracy.

With a CNN-based approach, the authors in [17] utilized the benchmark dataset of Brats 2013 for classifying brain tumors, thus achieving classification accuracy of 97%. In another study, the CNN-based method was applied to three distinct dataset, and after data augmentation using Deep CNN, the accuracy for meningioma, glioma, and pituitary tumor were 95%, 95%, and 98% respectively [18]. The “Tumour Cut” approach was proposed by Hamamci et al. [19]. This technique involved applying the algorithm to each MRI modality independently (e.g., T1, T2, T1-Gd and FLAIR). The overall tumor volume is then calculated by combining the results. Havaei et al. [20] applied a modern semi-automatic technique used with a fresh categorization strategy. A support vector machine (SVM) is trained to categorize all the voxels in an image to the appropriate tissue type using the intensity values and spatial coordinates extracted from these subsets of voxels as features.

Different CNN algorithms have been utilized for brain tumor classification as seen in Table 1, but not enough attention has been paid to low quality which may affect the classification performance of brain tumor and the problem of gradient vanishing due to the depth of the convolutional neural network. To this end, a novel parallelistic CNN model (PCNN) is proposed for brain tumor classification. The proposed model introduced CLAHE pre-processing algorithm for the removal of noisy artifact and enhancing the visible trainable features of the brain MRI images. Then, the meaningful details from the brain MRI images are extracted using the PCNN model during training and classified using sigmoid classifier to achieve higher identification accuracy. The strategy of parallelistic convolution neural network is proposed to take complete advantage of convolutions of multiple filters stacked in parallel to achieve wider network and ensure re-usability of visual features between parallelistic modules.

This paper focuses on the problem of low-quality brain tumor images. The novelties of our proposed model is in two-fold. First, a pre-processing technique is utilized to improve the inverse intensity and limit the histogram amplification of the image. Secondly, a parallelistic CNN model is proposed to learn distinctive features from the images using multiple filters of various sizes connected in parallel for the classification of brain tumor. A public dataset belonging to Kaggle is used to validate the performance of the proposed model.

## 3. Materials and Methods

The method adopted in this study is explained in this section. Data collection, data pre-processing, and the proposed networks for feature learning and classification are the main stages of this paper. The subsequent subsections of this section presents the procedure of the proposed method.

### 3.1. Datasets

**Figure 1:**
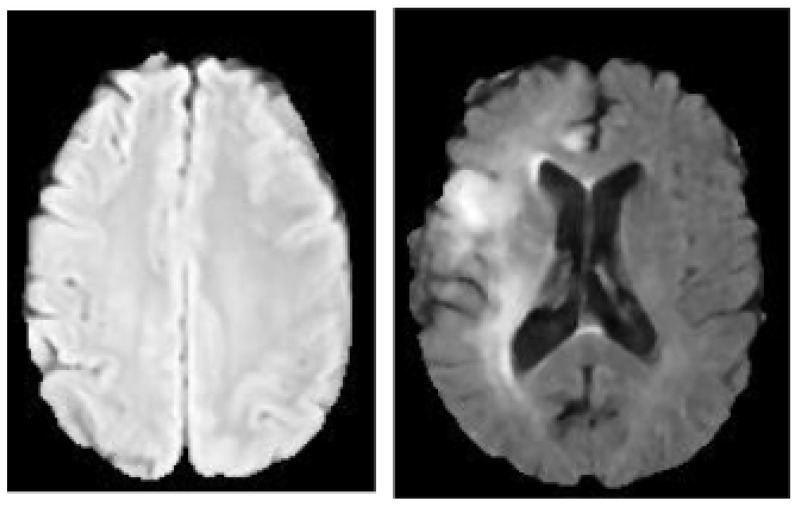
MRI brain images. The image on the left is Benign while the image on the right is Malignant.

The Kaggle Brain Tumor dataset is used for this study which includes two categories of brain tumor and healthy class [28]. This dataset is made up of 3762 MRI brain scan images of which 80% is utilized for training and 20% is used for testing to assess the model’s accuracy. The labels 0 indicates no tumor and 1 indicates the presence of tumor which is used to map the features onto the MRI images from the training dataset. Table 2 shows the MRI dataset from Kaggle database and Figure 1 shows the visual representation of the dataset.

### 3.2. Data Pre-Processing

As a matter of fact, data quality can be affected by noise, artifacts and resolution, hence, fitting such data directly to the CNN model may result to poor outcomes. Data pre-processing is introduced to eliminate or subside the noise and improve the quality of the data, thereby enhancing the performance of the algorithm. This study adopted an algorithm of Contrast Limited Adaptive Histogram Equalization (CLAHE) to pre-process the data.

#### CLAHE Images

**Figure 2:**
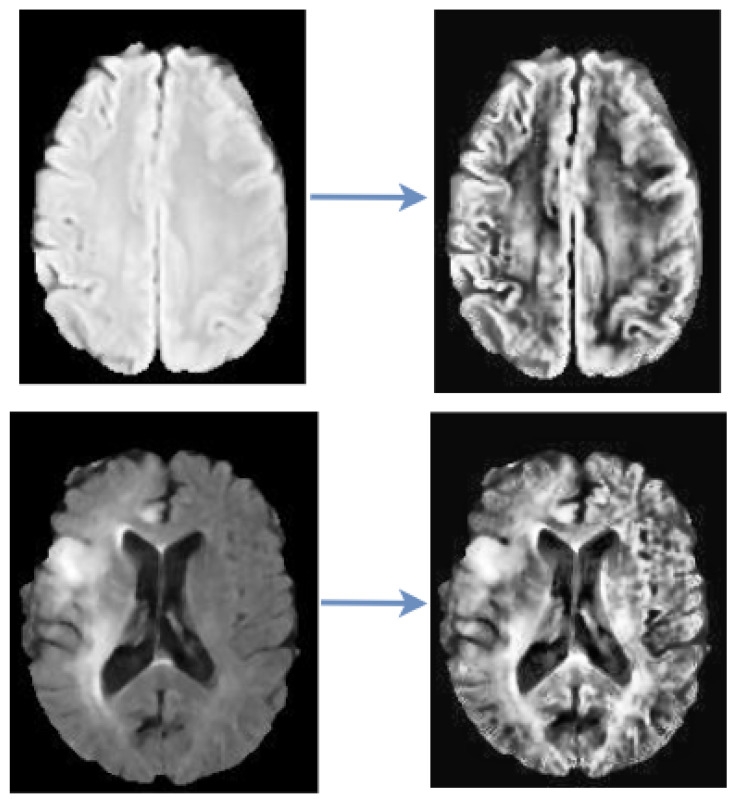
CLAHE Pre-processing. The images on the left are the raw images while the images on the right are the processed images.

This study utilized CLAHE as a pre-processing technique to enhance the contrast and features of the image by pronouncing abnormal patterns in the image. Contrast Limited Adaptive Histogram Equalization (CLAHE) algorithm produces a more realistic appearance among the histogram equalization family and it is capable of reducing noise amplification. We have examined the efficacy of CLAHE algorithm and adopted it to the MRI dataset, as shown in Figure 2.

### 3.3. Proposed Approach

For this study we propose a parallelistic Convolutional Neural Network. Convolutional Neural Network is an organized approach used in the processing various kind of images including medical images. A convolutional neural network (CNN) is a type of neural network that specializes in learning constituent knowledge and is used for image recognition. CNN is an effective image processing and computing technique that uses deep learning to carry out both generative and descriptive tasks. It typically makes use of machine vision, which has the ability to recognize images.

The proposed parallelistic convolution neural network (PCNN) involves learning filters of different sizes in a parallel manner to achieve a wider network instead of a deeper network. The proposed PCNN is made of 10 parallelistic modules divided into three blocks. Block A consists of three parallelistic modules of 1×1, 3×3, 1×3, 3×1 convolution layers while block B consists of five parallelistic modules of 1×1, 1×5, 5×1 convolution layers and finally, block C consists of two parallelistic modules of 1×1, 1×7, 7×1 convolution layers. The features learned from the filters of one parallelistic module are concatenated as output and passed to another parallelistic module as input within a block. Appearantly, the output of one parallelistic block becomes the input of the next parallelistic block with each block having its own unique filter sizes connected in parallel to ensure reusability of features and to avoid redundant learning. The input image has the size of 224×224×3 to match the proposed model design. The core structure of the proposed parallelistic convolution neural network model in this paper employs parallel convolution layers with multiple filters of various sizes.

**Figure 3:**
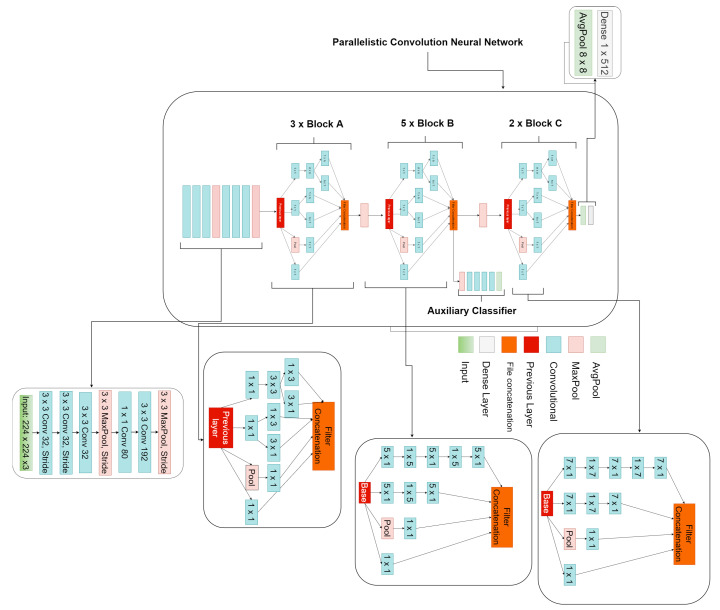
Framework of the proposed parallelistic convolution neural network.

The proposed model has a total of 57 convolution layer and 13 max-pool. The input image are feed into the separate stacked convolution layers of multiple filters from which the outputs are concatenated as a single output which becomes the new input to the proceding parallelistic blocks that provides a more robust representation for detecting the tumor.The intended technique is depicted in Figure 3. Then, to address the issue of vanishing gradients, we applied the relu activation function. The batch normalization method suggested by Sergey Ioffe et al in a 2015 study was then used [41].

In addition to producing neural networks more quickly and with greater stability, batch normalization was introduced to normalizes the inputs to the layers by re-centering and rescaling [34]. The pooling procedure entails applying a 2D filter to each channel of the feature map and aggregating the features that are present within the space of the filter. When a feature map with dimensions h×w is pooled, the result has the following dimensions as presented in Equation (1).

(1) $$\frac{\left(w\times f+1\right)}{s}\frac{\left(h\times f+1\right)}{s}$$

where *h* is the feature map heigth, *w* is the feature map width, *f* is filter size and *s* is the stride.

#### Features Extraction

The satisfactory performance of the propose parallelistic convolution neural network is due to the sevral network connectivity techniques, including batch normalization, using stacked convolutional layers of multiple filter sizes to replace linear convolutional layers, and factorizing convolutions to reduce dimensionality. These techniques considerably reduce the computational cost and number of network parameters, enabling the model to become wider instead of deeper as comapred to conventional CNN models.

The parallelistic convolution neural network model proposed in this paper is utilized on MRI brain dataset to perform tumor identification.

This study utilized an average pooling of 8×8 instead of the common dense layer to flatten the feature vector as seen in Figure 3.

Sigmoid is utilized to classify brain tumor images based on the feature vector and as a cost function, the binary cross entropy is utilized, given in Equation (2)

(2) $$L_{loss}=-\sum_{k=1}^{n}t_{k}\;log\;p_{k}$$

where *n* represents the number of class labels, $p_{k}$ is the instances for the k-th classes and $t_{k}$ is the corresponding label. The chance of event *k* occurring is $t_{k}$ meaning that the total sum of $t_{k}$ is 1, which implies that only one event is possible. The negative sign minimizes the loss when the distributions come closer to one other.

## 4. Results

This section presents thorough details of the experimental setup, performance metrics, performance of the proposed PCNN and comparison with other research methods.

### 4.1. Experimental Setup and Configuration

The proposed model is implemented on NVIDIA RTX 3060 GPU using Keras framework and tensorflow as backend. The entire dataset is split into 80:20 ratio for training and testing respectively. All the data are resized to 224×224 for both the raw and CLAHE MRI images. Adam optimizer and dropout are utilized with 0.0001 learning rate and batch size of 8. The proposed PCNN is trained separately on both the raw and CLAHE dataset. This study utilizes the Kaggle dataset of brain tumor. We captured a total of 3762 brain MRI images of various tumor kinds, including T1, T2, and FLAIR. This dataset consists of two classes; benign and malignant. Benign would mean non-tumor while malignant would mean tumor.

Table 3 demonstrate the outcomes of our experiments using the CLAHE pre-processing MRI images achieving 98.7% accuracy which is greater compared to the 94.7% accuracy obtained using the raw MRI images.

### 4.2. Performance Metrics

Accuracy, sensitivity, specificity, precision, and F1-score are the evaluation metrics utilized to validate the performance of the proposed PCNN. The mathematical representation for each metric is denoted in Equations (3)–(7).

(3) $$Accuracy=\frac{TP+TN}{TP+TN+FP+FN}$$

Accuracy depicts the percentage of correctly predicted classes.

(4) $$Sensitivity=\frac{TP}{TP+FN}$$

(5) $$Specificity=\frac{TN}{TN+FP}$$

Specificity measures the correctly predicted negatives cases.

(6) $$Precision=\frac{TP}{TP+FP}$$

(7) $$F1-score=2*\frac{Precision*Recall}{Precision+Recall}$$

$F_{1}$ score gives the mean of precision and recall as stated in Equation (7). Recall depicts the proportion of correctly predicted positives classes. Precision depicts how accurate the model makes predictions.

The Reciever Operation Curve (ROC) shows the relationship between sensitivity against 1−specificity. AUC is a widely adopted metric to measure the overall accuracy of the model.

TP, FP, FN, and TN represent true positive, false positive, false negative, and true negative, respectively.

### 4.3. Evaluation of the Proposed Model

On the brain dataset, we conducted two experiments to validate the efficacy of the proposed model. The first investigation is implementing the proposed PCNN on the raw brain dataset without pre-processing. Secondly, we implemented the proposed model on the CLAHE pre-processed brain dataset for brain tumor identification.

**Figure 4:**
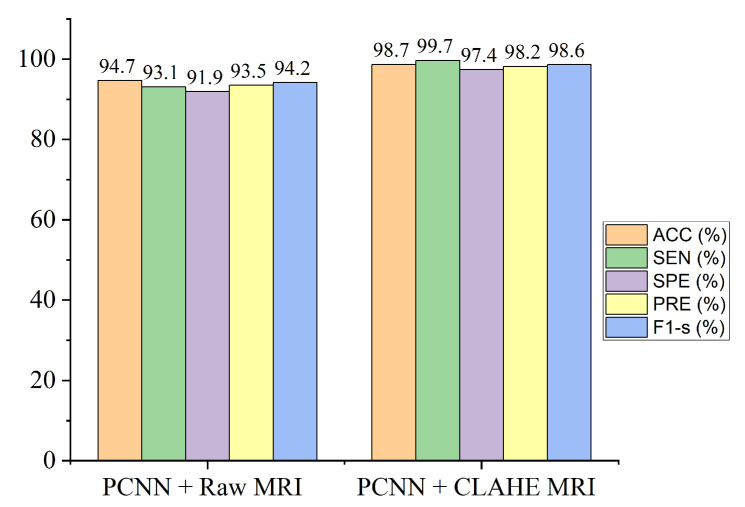
Performance of the proposed PCNN in comparison with raw MRI images and CLAHE pre-processed MRI images across the different evaluation metrics.

The proposed framework of the parallelistic convolution neural network as shown in Figure 3, clearly shows that the pre-processed image features has the ability to manage low-quality images in brain tumor diagnosis, attaining enhanced performance accuracy on the brain dataset. Figure 4 shows classification performance of the proposed model for both the raw and pre-processed MRI across all the evaluation metrics. It is obvious that the proposed PCNN with the pre-processed dataset achieves better results than the raw dataset by a considerable margin.

### 4.4. Result of the Proposed Model

**Figure 5:**
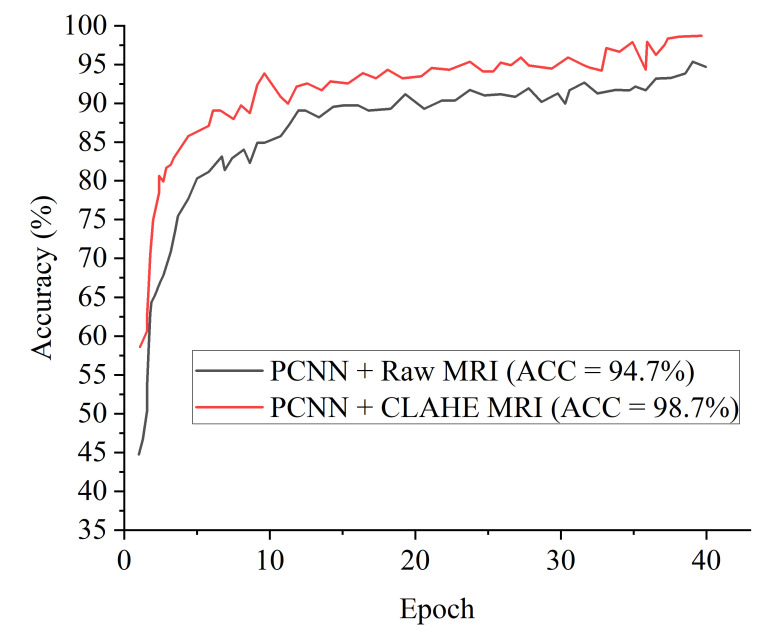
Accuracy curves for the proposed PCNN in comparison with raw MRI images and CLAHE pre-processed MRI images.

**Figure 6:**
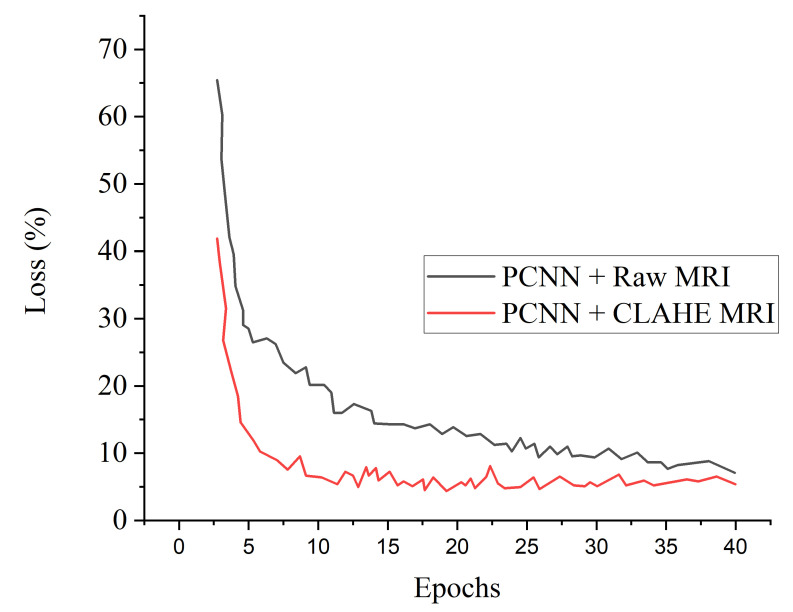
Loss curves for the proposed PCNN in comparison with raw MRI and CLAHE pre-processed MRI images.

**Figure 7:**
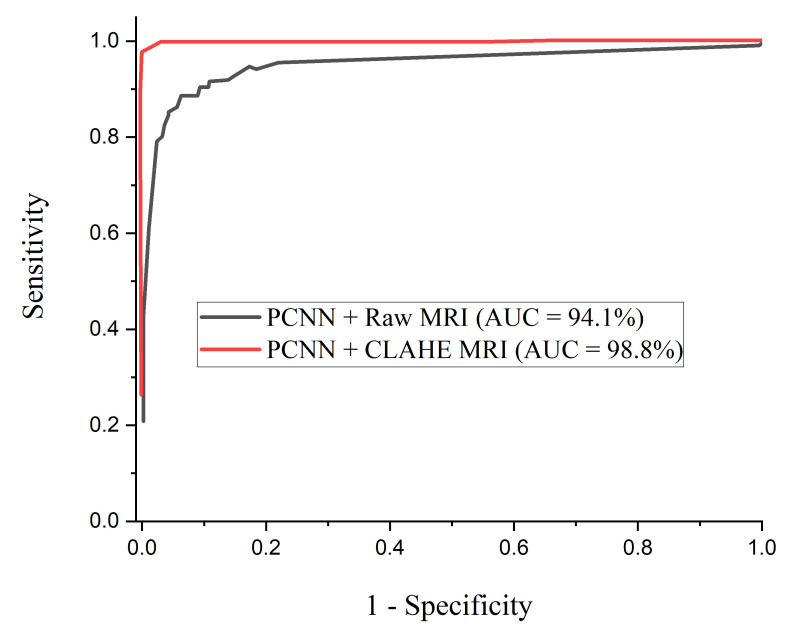
ROC curves for the proposed PCNN in comparison with raw MRI and CLAHE pre-processed MRI images.

Figure 5 denotes the accuracy curves for the proposed PCNN for both the raw and CLAHE pre-processed MRI. The first few epochs shows the accuracy quickly increases to about 85% for the proposed PCNN on the CLAHE pre-processed MRI and then progresses gradually. Figure 6 presents the loss curves showing gradual reduction in loss for the proposed PCNN model for both the raw and CLAHE pre-processed MRI. Figure 7 depcts the ROC-AUC curves for the proposed PCNN model for both the raw and CLAHE pre-processed MRI. The PCNN Raw represents the PCNN model implemented on raw MRI dataset for the identification of brain tumor with 94.1% AUC. The PCNN CLAHE represents the PCNN model implemented on CLAHE pre-processed MRI dataset with 98.8% AUC.

**Figure 8:**
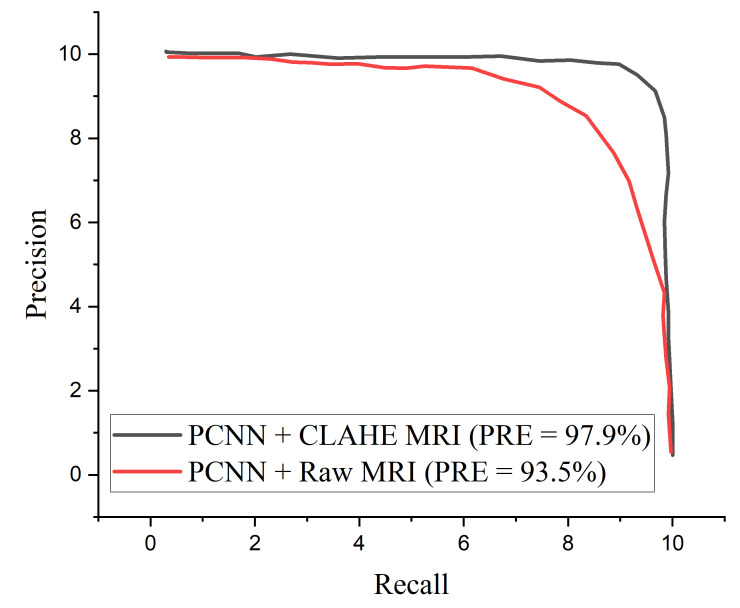
Precision–recall curves for the proposed PCNN in comparison with raw MRI and CLAHE pre-processed MRI images.

The proposed PCNN model shows satisfactory performance when implemented on CLAHE pre-processed MRI images compared to the raw MRI images. The proposed PCNN model was further evaluated in terms of precision–recall curve, as depicted in Figure 8. As the curve tends toward the upper right hand corner of the graph, it is obvious that the proposed PCNN using CLAHE pre-processed MRI images Performs satisfactorily compared to using the raw MRI images, indicating that the model has high precision associated with high recall.

## 5. Discussion

The performance of the proposed model in diagnosing brain tumor in MRI images from dataset obtained Kaggle repository has been demonstrated, and the classification result is presented in Table 3. The proposed PCNN model can effectively identify distinct tumor from healthy MRI images. It is worth mentioning that the CLAHE pre-processed MRI images result in a greater generalization ability for the proposed PCNN model achieving 98.7% accuracy, 99.7% sensitivity, 97.4% specificity, 98.2% precision and 98.6% f1-score.

**Figure 9:**
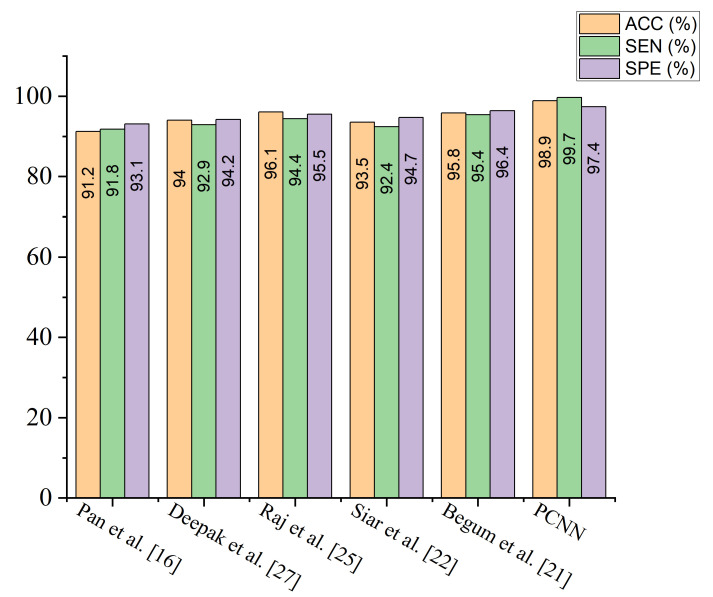
Performance of the proposed PCNN in comparison with some selected state of the art models using the same dataset.

A comparison study is conducted between the proposed PCNN model and some recently published brain tumor classification methods. According to Table 4, the proposed PCNN model obtained the highest accuracy and sensitivity score of 98.9% and 99.7%, respectively, demonstrating its superiority in the identification of brain tumor while Siar et al. [27] achieve the highest specificity score of 100%. The integration of CLAHE pre-processing of the MRI images prior to model training gives the proposed technique a competitive advantage. For the sake of fair comparison, some state-of-the-art brain tumor methods are selected and implemented on the same MRI dataset using the same computing resources as presented in Table 5. It is worth mentioning that different deep learning algorithms will behave differently owing to various situations. More so, few recent state-of-the-art deep transfer learning models are selected and a fair comparison is conducted using the same dataset and computing resources as depicted in Table 6. Figure 9 shows the performance of the selected few state-of-the-art brain tumor methods across various matrices using the same MRI data.

According to the results of the experiments as presented in Table 6, the proposed PCNN model outweighs the deep transfer learning models across all evaluation metrics. Inception-V3 achieves slightly higher results on CLAHE pre-processed in terms of accuracy and sensitivity compared to the raw MRI images but obtained a slightly lower result in specificity of 90.2%. The other pre-trained models consistently achieved higher result on the CLAHE pre-processed MRI images compared to the raw MRI images which indicates that the CLAHE pre-processing algorithm is capable of eliminating noise and enhancing the visual trainable feature of the MRI images. More so, the computational complexity of the proposed model is far reduced compared to the other methods as depicted in Table 5 and Table 6 which show the running time of the proposed model and other methods including popular deep learning models.

Additionally, the satisfactory performance of the proposed PCNN can be attributed to our technique of stacking convolution layers with multiple filters of different sizes in parallel. The advantage of the multiple filters of different sizes is to achieve a wider receptive field to learn distinctive features without just memorizing the patterns which is limited in regular deep learning models which only utilizes one filter size at each convolution layer stacked vertically. We also introduce bottleneck technique to shrink the feature maps by using 1×1 convolution. Another important component we utilized is the factorization technique where we utilized 1×3 and 3×3 filter sizes to replace 3×3 filter size in order to reduce the computation complexity and accelerate the training time.

**Figure 10:**
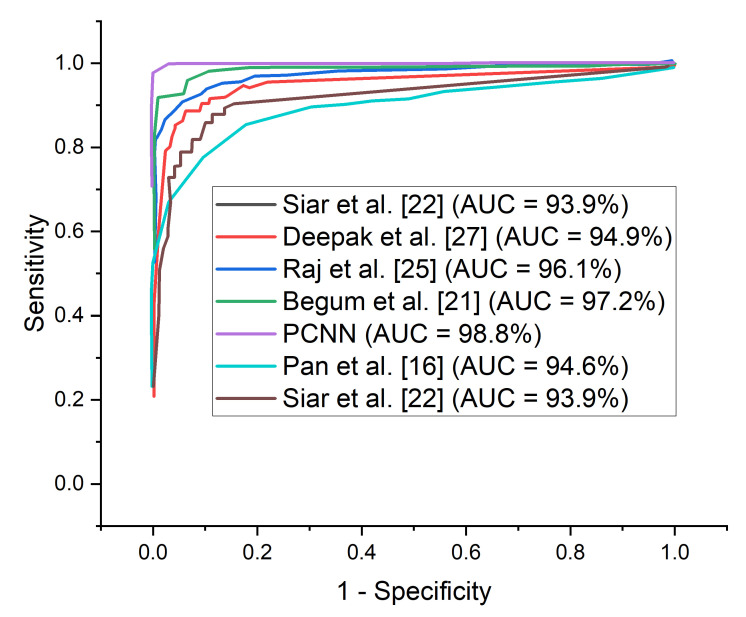
ROC-AUC curves for the proposed PCNN in comparison with some selected state of the art models using the same dataset.

**Figure 11:**
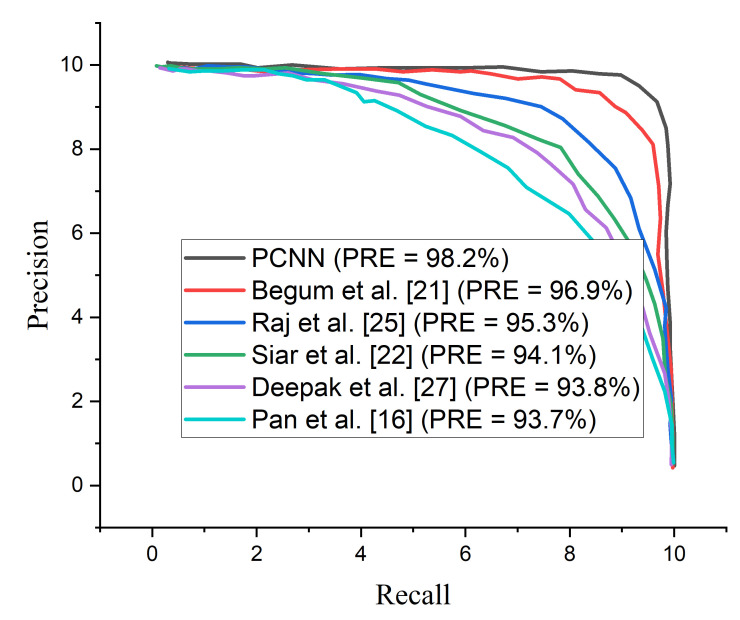
Precision–Recall curves for the proposed PCNN in comparison with some selected state of the art models using the same dataset.

It is important to adopt the ROC curve to measure the overall accuracy (AUC) as well as the precision–recall curve to estimate the average precision of the proposed PCNN model when classifying delicate conditions like brain tumor. The ROC curves for the proposed PCNN model on the brain dataset is depicted in Figure 10. Similarly, Figure 11 represents the precision–recall curve for the proposed PCNN model.

More so, many of the brain MRI images were blurry and lacking in features, which may have hindered the ability of the proposed PCNN to obtain and train relevant characteristics. The advantage of adopting CLAHE pre-processing algorithm is to enhance the low quality of the brain MRI images in order to identify high representation features of the MRI images with visible trainable characteristics. The proposed PCNN model performed admirably in identifying brain tumor.

In terms of ROC and precision–recall curves, the proposed PCNN model outperforms the state-of-the-art methods for brain tumor classification, especially when dealing with low-quality brain MRI images. The curve of the proposed PCNN is nearest to the upper left corner of the graph and has the highest area under curve, depicting that it has higher sensitivity associated with high specificity according to the ROC graph in Figure 10. Also, the curve of the proposed PCNN is nearest to the upper right corner of the graph, indicating that it has higher precision associated with higher sensitivity in Figure 11. The experimental results stated in terms of Receiver Operating Characteristic (ROC) and precision–recall can aid neurosurgeon to strike a balance between precision and accuracy.

Nevertheless, other state-of-the-art methods have shown considerable performance in brain tumor classification, however, the researchers did not take into consideration the low quality of brain MRI images which is a concern in real-life application. It is a known fact that the quality of data can be affected by noise, resolution, and artifacts. The direct application of such data in an algorithm may affect the adaptability of the model in clinical application. Data pre-processing could help in mitigating the noise and improving the data quality, thereby enhancing the performance of the model.

### 5.1. Ablation Study

#### Hyperparameter Tuning

Ablation study is conducted to examine if hyper-parameter tuning may improve the performance of the proposed parallelistic convolution neural network. In this study, we considered both the raw MRI images and the pre-processed images. We term the unpre-processed MRI images as RAW and the pre-processed MRI images as CLAHE. The results of different optimizers at different learning rates and dropouts are shown in Table 7, Table 8 and Table 9. The proposed parallelistic convolution neural network obtains the best performance utilizing Adam optimizer with a learning rate of 0.0001 and dropout of 0.50, attaining 98.7% accuracy, according to Table 7. It is worth mentioning that the Adam optimizer is substantially more robust than the other optimizers (RMSProp and SGD) due to its computational efficiency.

## 6. Conclusions

In this study, we proposed a parallelistic convolutional neural network (PCNN) with Contrast Limited Adaptive Histogram Equalization (CLAHE) algorithm with the aim of addressing the vanishing gradient problem of deeper convolutional neural network and low quality in MRI brain images respectively. We implemented the CLAHE pre-processing algorithm for the removal of noisy artifact and enhancing the visible trainable features of the brain MRI images. Then, the meaningful details from the brain MRI images are extracted using the PCNN model during training and classified using sigmoid classifier to achieve higher identification accuracy. The strategy of parallelistic convolution neural network is proposed to take complete advantage of convolutions of multiple filters stacked in parallel to achieve wider network and ensure re-usability of visual features between Parallelistic modules.

Furthermore, average pooling was introduced to vectorize the feature embeddings instead of the conventional fully connected layer before the Sigmoid classifier to obtain predictions. By incorporating convolution layers of multiple layers stacked in a parallel manner, the proposed model outperforms several deep transfer learning models and state-of-the-art brain tumor methods. The evaluation results illustrate that the proposed PCNN obtains satisfactory performance with accuracy of 98.7%, sensitivity of 99.7%, specificity of 97.4%, precision of 98.2%, and f1-score of 98.6% than just utilizing the raw MRI brain images. In terms of comparison with the other established models, it is confirmed that the proposed PCNN model obtained state-of-the-art classification performance, which is a robust and efficient classification solution for brain tumor based on low quality MRI images. These experimental outcomes could effectively assist neurosurgeons and other clinicians to diagnose what stage of brain tumor is present in a patient’s MRI brain scan while preserving screening time.

## 7. Limitation and Future Work

From the experimental analysis, the proposed PCNN achieved excellent results across all evaluation metrics. In this research, we focused more on improving the existing CNN models that has been proposed for classifying brain tumor using the same dataset from Kaggle repository. The proposed model only considered a balanced class of dataset with just two categories of tumor classification which is the limitation of this study. In the future, we will consider imbalance class of dataset and multiple categories of tumor class and also take into account different disease severity.More so, we intend to improve the robustness of the model by introducing a point-wise separable convolution in order to reduce the network weight which would make the model more computational efficient.

## Figures and Tables

**Table 1:** Summary of the Related Works.

Authors	Year	Dataset	Techniques	Evaluation Results
Pan et al. [21]	2020	Fig-share Images	CNN	ACC = 92%
Maharjan et al. [22]	2019	Fig-share Images	Residual Network ResNet	ACC = 95%
Vimal et al. [23]	2019	KaggleTCIA	PNN Classification CNN	ACC = 90%
Boustani et al. [24]	2020	Kaggle	CNN	ACC = 99%
Mukherkjee et al. [25]	2019	Pvt Dataset	R-CNN and SVM	ACC = 95%
Begum et al. [26]	2020	Fig-share Images	ELM-LRF CNN	ACC = 97%
Siar et al. [27]	2020	BRATS 13,14,17,18	Inception Pre-trained CNN	ACC = 92%
Anilkumar et al. [28]	2019	Kaggle	CNN	ACC = 99%
Ari et al. [29]	2019	BRATS 2016	ResNet-50 for Detection Gan for Data Augmentation	ACC = 92%
Raj et al. [30]	2020	Private Dataset Comprising of 1000 images	RNN	ACC = 96%
Joshi et al. [31]	2019	Private Dataset Comprising of 330 images	CNN	ACC = 98%
Deepak et al. [32]	2020	BRATS CE-MRI	VGGnet and KNN as Classifier	ACC = 98.69%
Krishnammal, P. et al. [33]	2019	Fig-share Images	CNN	ACC = 96%
Chattopadhyay et al. [34]	2020	(BITE), Fig share (4689 detection and for classification	BRAIN NETs for Detection and Classification	ACC = 98%
Poonguzhali, N. et al. [35]	2019	BRATS 2018	3D-Multi CNNs	ACC = 84%
Kachwalla et al. [36]	2018	Fig-Share and REMBRA NDT 516 Images	CNN for Grading and Classification	ACC = 96%
Han et al. [37]	2019	BRATS 2015	AlexNet and VGG-16	ACC = 98%
Mohsen et al. [38]	2018	Harvard Dataset (66 MRIs with 22 and 44 standard images vs. affected	DNN for Classification and Fuzzy-C for Segmentation	ACC = 98%
Krishnammal et al. [33]	2016	1000 Private Images from Indian Hospital	DNN and ELM	ACC = 96%
Athency et al. [39]	2017	BRATS 2015	3D CNN	ACC = 75.4%
Pandian et al. [40]	2017	BRATS 2017 and TCIA	ConvNet, SliceNet and VGNet	ACC = 97%

**Table 2:** Description of the Dataset.

Dataset	Brain Tumor Category	Value
Kaggle database of Brain Tumor [28]	Benign	2079
	Malignant	1683

**Table 3:** Performance evaluation of the proposed parallelistic convolution neural network using the raw MRI and pre-processed CLAHE MRI images.

Model	ACC (%)	SEN (%)	SPE (%)	PRE (%)	F1-s (%)	Time (min)
PCNN + CLAHE MRI images	98.7	99.7	97.4	98.2	98.6	6.57
PCNN + Raw MRI images	94.7	93.1	91.9	93.5	94.2	6.81

**Table 4:** Result comparison of the proposed PCNN model with state-of-the-art methods for brain tumor classification.

Authors	ACC (%)	SEN (%)	SPE (%)
Pan et al. [21]	92.0	91.0	91.0
Siar et al. [27]	92	90.9	100.0
Chattopadhyay et al. [34]	98.0	92.9	98.8
Deepak et al. [32]	98.6	86.9	-
Raj et al. [30]	96.0	-	-
Begum et al. [26]	97.0	99.6	-
Boustani et al. [24]	99.0	79.2	90.7
Vimal et al. [23]	90.0	90.7	95.5
Han [37]	98.0	96.1	95.7
Maharjan et al. [22]	95.0	64.7	92.9
PCNN + CLAHE	98.7	99.7	97.4

**Table 5:** Comparison table for the selected state of the art models using the same dataset and computing resource.

Model	ACC (%)	SEN (%)	SPE (%)	Time (min)
Pan et al. [21]	91.2	91.8	93.1	11.38
Deepak et al. [32]	94.0	92.9	94.2	8.07
Raj et al. [30]	96.1	94.4	95.5	15.72
Siar et al. [27]	93.5	92.4	94.7	10.84
Begum et al. [26]	95.8	95.4	96.4	13.71
PCNN + CLAHE	98.7	99.7	97.4	6.57

**Table 6:** Results obtained using different pre-trained models in comparison with the proposed PCNN on the same dataset.

	RAW MRI Imagesl			CLAHE MRI Images				
Model	**ACC (%)**	**SEN (%)**	**SPE (%)**	**Time (min)**	**ACC (%)**	**SEN (%)**	**SPE (%)**	**Time (min)**
Inception-V3	89.2	91.4	92.6	9.46	89.3	87.5	90.2	9.51
MobileNet-V3	88.9	90.7	91.4	7.41	90.9	90.1	91.8	7.49
ResNet-152	84.6	86.2	87.9	10.13	91.4	92.3	93.1	10.20
VGG-16	87.7	89.4	90.5	11.89	93.7	95.4	93.1	11.90
DenseNet-121	85.3	87.1	88.7	11.74	92.8	92.8	93.3	11.70
AlexNet	86.3	88.6	89.4	6.58	93.1	91.5	93.7	6.51
PCNN	94.7	93.1	93.9	6.81	98.7	99.7	97.4	6.57

**Table 7:** Performance evaluation of the proposed PCNN based on different hyper-parameter tuning on our dataset with Adam optimizer.

Hyperparameters	(PCNN + CLAHE + Adam)	(PCNN + RAW + Adam)
	Accuracy (%)	Accuracy (%)
LR (0.1) + Dropout (0.25)	87.5	81.6
LR (0.1) + Dropout (0.50)	86.9	87.3
LR (0.1) + Dropout (0.75)	83.6	82.6
LR (0.01) + Dropout (0.25)	81.4	85.1
LR (0.01) + Dropout (0.50)	89.8	84.9
LR (0.01) + Dropout (0.75)	84.7	90.7
LR (0.001) + Dropout (0.25)	82.2	88.2
LR (0.001) + Dropout (0.50)	80.7	91.3
LR (0.001) + Dropout (0.75)	88.3	92.7
LR (0.0001) + Dropout (0.25)	85.9	83.4
LR (0.0001) + Dropout (0.50)	98.7	94.7
LR (0.0001) + Dropout (0.75)	79.5	86.2

**Table 8:** Performance evaluation of the proposed PCNN based on different hyperparameter tuning on our dataset with RMSProp optimizer.

Hyperparameters	(PCNN + CLAHE + RMSProp)	(PCNN + RAW + RMSProp)
	Accuracy (%)	Accuracy (%)
LR (0.1) + Dropout (0.25)	88.5	89.1
LR (0.1) + Dropout (0.50)	89.7	87.4
LR (0.1) + Dropout (0.75)	86.3	90.7
LR (0.01) + Dropout (0.25)	87.7	88.2
LR (0.01) + Dropout (0.50)	81.4	91.5
LR (0.01) + Dropout (0.75)	81.1	89.8
LR (0.001) + Dropout (0.25)	83.8	92.3
LR (0.001) + Dropout (0.50)	86.5	89.6
LR (0.001) + Dropout (0.75)	84.2	90.9
LR (0.0001) + Dropout (0.25)	85.9	91.1
LR (0.0001) + Dropout (0.50)	88.6	89.5
LR (0.0001) + Dropout (0.75)	85.3	88.9

**Table 9:** Performance evaluation of the proposed PCNN based on different hyperparameter tuning on our dataset with SGD optimizer.

Hyperparameters	(PCNN + CLAHE + SGD)	(PCNN + RAW + SGD)
	Accuracy (%)	Accuracy (%)
LR (0.1) + Dropout (0.25)	87.2	88.8
LR (0.1) + Dropout (0.50)	85.5	89.3
LR (0.1) + Dropout (0.75)	87.9	97.5
LR (0.01) + Dropout (0.25)	89.1	90.9
LR (0.01) + Dropout (0.50)	82.3	92.3
LR (0.01) + Dropout (0.75)	83.6	88.7
LR (0.001) + Dropout (0.25)	84.9	91.9
LR (0.001) + Dropout (0.50)	85.7	90.4
LR (0.001) + Dropout (0.75)	83.5	91.6
LR (0.0001) + Dropout (0.25)	86.3	89.3
LR (0.0001) + Dropout (0.50)	87.1	90.8
LR (0.0001) + Dropout (0.75)	88.8	89.2

## Data Availability

The dataset used in this study can be found in the followig link: https://www.kaggle.com/datasets/jakeshbohaju/brain-tumor accessed on: 13 May 2022.

## Acronym/Abbreviations

The following abbreviations are used in this manuscript:

PCNN	Parallelistic Convolution Neural Network
ML	Machine Learning
DL	Deep Learning
TL	Transfer Learning
CNN	Convolutional Neural Network
ReLu	Rectified Linear Unit
SGD	Stochastic Gradient Descent
LR	Learning Rate
Acc	Accuracy
F1	F1-score
Pre	Precision
Rec	Recall
Spe	Specificity
TP	True Positives
TN	True Negatives
FP	False Positives
FN	False Negatives
ROC	Receiver Operation Curve
AUC-ROC	Area Under the Receiver Operation Curve
